# A girl with protein-losing enteropathy during a ketogenic diet: a case report

**DOI:** 10.1186/s12887-020-1991-8

**Published:** 2020-03-03

**Authors:** Juan Wang, Li Jiang, Min Cheng

**Affiliations:** Department of Neurology, Ministry of Education Key Laboratory of Child Development and Disorders, National Clinical Research Center for Child Health and Disorders, China International Science and Technology Cooperation base of Child development and Critical Disorders, Chongqing Key Laboratory of Translational Medical Research in Cognitive Development and Learning and Memory Disorders, Children’s Hospital of Chongqing Medical University, No.136, zhongshan 2nd road, yuzhong district, chongqing, 400014 China

**Keywords:** Ketogenic diet, Protein-losing Enteropathy, Edema, Intractable epilepsy

## Abstract

**Background:**

A ketogenic diet (KD) is an effective treatment for intractable epilepsy in children. Protein–losing enteropathy (PLE) is a rarely reported but serious complication of KDs.

**Case presentation:**

A 3-month-old female patient presented with PLE while following a KD as treatment for intractable epilepsy. She also had genovariation of the STXBP1 gene. The patient suffered from general edema and hypoalbuminemia but no diarrhea. Esophagogastroduodenoscopy (EDG) revealed lymphatic ectasia in the lamina propria. We diagnosed her with intestinal lymphangiectasia, and after decreasing the KD ratio from 4:1 to 1.05:1, we successfully controlled her edema and hypoalbuminemia. As of now, the convulsions and hypsarrhythmia have disappeared, and the seizure-free state has lasted for 20 months.

**Conclusions:**

PLE may be managed by decreasing the ketogenic ratio rather than discontinuing a KD since for some patients, a KD is the only effective therapy available at present.

## Background

A ketogenic diet (KD) is an effective treatment for intractable epilepsy in children. Protein–losing enteropathy (PLE) is a rarely reported but serious complication of KDs [1, 2]. A female infant with genovariation of the STXBP1 gene suffered multiple types of seizures, including tonic seizures, epileptic spasms, focal seizures, and tonic-clonic seizures. Her CT scan and MRI showed no abnormalities, but her EEG was characterized by hypsarrhythmia. She was treated with levetiracetam prednisone, nitrate diazepam, topiramate, and a ketogenic diet (KD). When the patient was 3 months old, she suffered protein–losing enteropathy (PLE) while following the KD. She suffered from general edema and hypoalbuminemia but no diarrhea. Esophagogastroduodenoscopy (EDG) revealed lymphatic ectasia in the lamina propria, and we diagnosed her with intestinal lymphangiectasia. After decreasing the KD ratio from 4:1 to 1.05:1, we successfully controlled her edema and hypoalbuminemia. As of now, the convulsions and hypsarrhythmia have disappeared, and the patient has been seizure-free for 20 months.

A female infant with genovariation of the STXBP1 gene suffered multiple types of seizures, including tonic seizures (originating from the second day after birth), epileptic spasms and focal seizures (originating from the neonatal period), and tonic-clonic seizures (originating from the age of 2 months). Despite treatment with multiple antiepileptic drugs (AEDs), including levetiracetam prednisone, nitrate diazepam, and topiramate, the frequency of convulsions continued to increase, and her epileptic seizures even caused mental regression. The patient’s CT scan and MRI showed no abnormalities, but her electroencephalogram (EEG) shifted from multifocal discharges to hypsarrhythmia.

The patient was diagnosed with infantile spasms (IS) with multiple types of seizures, including tonic seizures, epileptic spasms, focal seizures, and tonic-clonic seizures. She also experienced mental and physical retardation after the seizures, such as the inability to follow sound or light and the loss of body movement. Her EEG was characterized by hypsarrhythmia, but her MRI showed no abnormalities. The patient had been treated with 4 AEDs for 3 months, and the seizures were still progressing, which indicated that the epilepsy was medically refractory.

At the age of 3 months, the patient’s urine organic acids, serum amino acids, complete blood count, serum liver and kidney tests, and abdominal B-scan ultrasound results were all normal. Therefore, we treated her with a KD.

Initially, we treated her with a KD with a 2:1 ratio of fat to nonfat according to the milk formula (Zeneca Biological Technology Company, China) and monitored her blood ketones regularly. Multiple AEDs were also maintained at the same doses. After 1 week of the KD, because her blood ketone levels were very low (average 1.5 mmol/L) and the seizures continued, we gradually increased the KD ratio to 4:1. Subsequently, the seizure frequency decreased. At the end of the first month on the KD, a seizure-free state was reached, and there was abatement of other complications such as vomiting and diarrhea (blood ketone: 1.9–3.3 mmol/L, glucose: 3.9–5.9 mmol/L).

In the second month of the KD, we found that the patient’s albumin levels decreased to 24.2 g/L when she suffered severe pneumonia, but she did not have edema. She was treated with intravenous albumin (blood ketone: 3.2–4.5 mmol/L, glucose: 4.4–5.1 mmol/L).

**Fig. 1:**
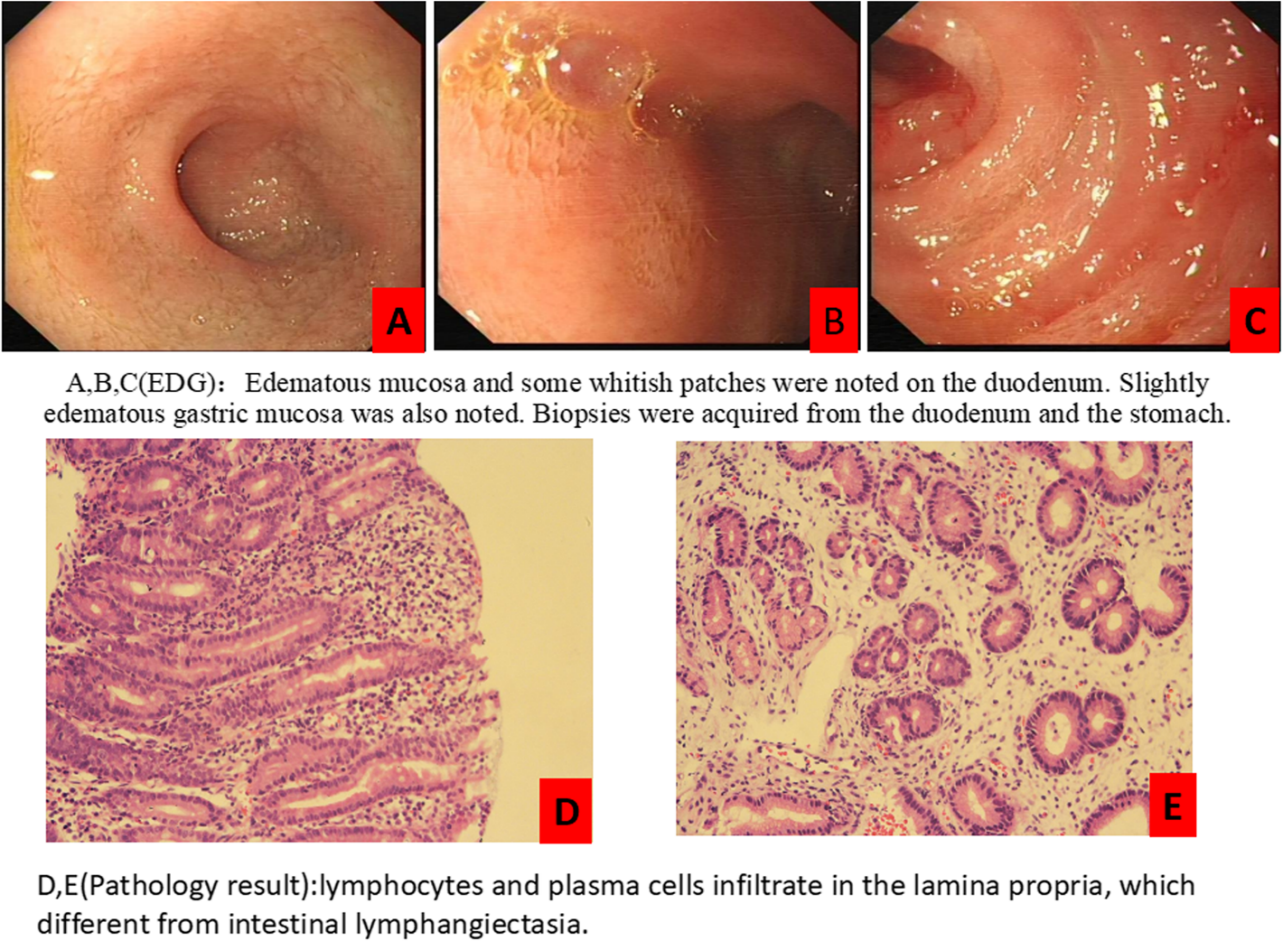
The change of Esophagogastroduodenoscopy: **a,b,c** (EDG):Edematous mucosa and some whitish patches were noted on the duodenum. Slightly edematous gastric mucosa was also noted. Biopsies were acquired from the duodenum and the stomach. **d**, **e** (Pathology result):lymphocytes and plasma cells infiltrate in the lamina propria, which different from intestinal lymphangiectasia)

In the third month of the KD, the patient’s albumin levels decreased significantly to 28.5 g/L, and she suffered from edema (blood ketone: 4.3–7.7 mmol/L, glucose: 4.5–5.5 mmol/L). We found no common causes of protein loss in the skin, urine, and blood tests; echocardiography; stool screening for pathogens; and abdominal ultrasonography. Her EGD (Fig. 1) revealed edematous mucosa in the duodenum, and the biopsy results revealed lymphocytes and plasma cells infiltrating the lamina propria, which was different from lymphatic ectasia. These findings were consistent with intestinal lymphangiectasia as a presentation of PLE. Thus, we diagnosed the patient with PLE [3] and treated her with intravenous albumin. However, her blood ketone levels were very high—up to 6–8 mmol/L—and therefore we gradually decreased the KD ratio to 1.05:1, in accordance with her blood ketone values. After 2 weeks of this treatment, the hypoalbuminemia and edema were resolved (blood ketone: 2.9–3.3 mmol/L, glucose: 5.1–5.2 mmol/L, albumin: 37.6 g/L), and the patient’s general condition was improved.

**Fig. 2:**
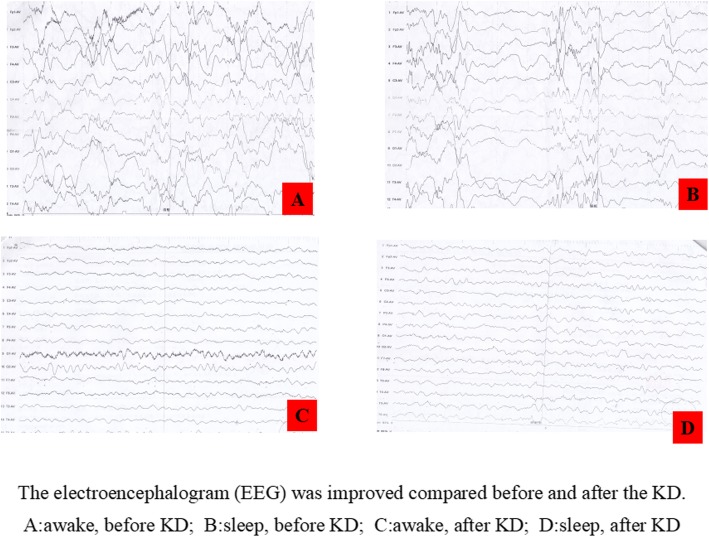
The electroencephalogram (EEG) was improved compared before and after the KD. **a**: awake, before KD; **b**: sleep, before KD; **c**: awake, after KD; **d**: sleep, after KD

At the end of the 4th month on the KD, the patient’s albumin level was 38.9 g/L, and she remained seizure-free. Furthermore, as of now, the seizure-free status has lasted for 20 months. The latest albumin level, tested on February 23, 2018, was 40.4 g/L. Meanwhile, her electroencephalogram (EEG) also improved (Fig. 2), and the hypsarrhythmia disappeared. In the follow-up clinic visits, we found no side effects on height, weight, BMI, blood tests, urine tests, abdominal ultrasonography, hepatic and renal functions, or microelements in the patient.

## Discussion and conclusions

PLE is a rarely reported but serious complication of a KD. Although hypoproteinemia is one of the presentations of PLE, it is also much more common than PLE [1, 2]. There are two published case reports for PLE initiated by a KD [4, 5], in both of which the patients discontinued the KD. Our patient is the first to not discontinue a KD when suffering from PLE. Our results suggest that decreasing the ketogenic ratio may be a feasible way of managing PLE.

The main mechanisms of PLE are mucosal injury and lymphatic abnormalities [3], and lymphangiectasia is suspected to be a mechanism of PLE [4, 5]. Intestinal lymphangiectasia has been reported in children following a high-fat diet [3] and may cause the leakage of lymphatic fluids rich in albumin and other proteins into the GI tract. Our patient had no history of intestinal disease and showed hypoproteinemia and intestinal lymphatic changes after starting the KD. This suggested that the intestinal lymphangiectasia was secondary to the KD.

The primary treatments of PLE include the maintenance of nutritional status and treatment of the underlying disease. Hypoproteinemia can be improved by increasing the protein intake, which requires a reduction of the ketogenic ratio. We therefore decreased the ratio of lipids in the KD, which lead to an improvement in the hypoproteinemia and patient condition, as expected.

Our results suggest that instead of discontinuing a KD, decreasing the ketogenic ratio may be a feasible method of managing PLE. After all, for some patients, a KD is the only effective therapy available at present. However, our study is limited by the failure to conduct a stool alpha-1-antitrypsin concentration test. Which KD patients are susceptible to PLE and which PLE patients can or cannot benefit from lowering the ratio of KD remain to be determined through further studies.

## Data Availability

Not applicable.

## Abbreviations

AEDs: Antiepileptic drugs
EDG: Esophagogastroduodenoscopy
EEG: Electroencephalogram
IS: Infantile spasms
KD: Ketogenic diet
PLE: Protein–losing enteropathy
